# Assessing Allostatic Load in Ring-Tailed Lemurs (*Lemur catta*)

**DOI:** 10.3390/ani11113074

**Published:** 2021-10-28

**Authors:** Kathryn E. Seeley, Kathryn L. Proudfoot, Barbara Wolfe, Douglas E. Crews

**Affiliations:** 1 The Columbus Zoo & Aquarium, Powell, OH 43065, USA; 2 Health Management, Atlantic Veterinary College, University of Prince Edward Island, Charlottetown, PE C1A 4P3, Canada; kproudfoot@upei.ca; 3 Clinical Sciences, College of Veterinary Medicine and Biomedical Sciences, Colorado State University, Fort Collins, CO 80523, USA; Barb.Wolfe@colostate.edu; 4 Department of Anthropology, The Ohio State University, Columbus, OH 43210, USA; crews.8@osu.edu

**Keywords:** allostatic load index, chronic stress, stressors, non-human primates, *Lemur catta*

## Abstract

**Simple Summary:**

Our ability to measure and understand chronic stress in animals is limited by animals’ inability to communicate to us their perception of stressors. Researchers have developed tools to measure stress in animals, but these tools have many limitations. A novel measurement of chronic stress using several biomarkers has been studied in humans and great apes, but no research has assessed this tool in other primates, including lemurs. Our aim was to determine the relationship between allostatic load index (ALI), a measurement of chronic stress, and individual, social, medical and husbandry factors that may impact stress in lemurs housed under human care. Smaller group sizes, a higher percentage of time spent indoors, and more frequent group changes were associated with a higher ALI, indicating that these factors may be associated with chronic stress and potential deleterious health effects. This research presents support for ALI as a tool to help us better understand and mitigate stress in animals under our care.

**Abstract:**

Responses to stress are unavoidable, adaptive mechanisms in humans and non-human animals. However, in humans, chronic stress has been linked to poor health outcomes and early mortality. Allostatic load, the physiologic dysregulation that occurs when an organism is exposed to chronic stressors, has been used to assess stress in humans; less work has been done using non-human primates. Our aim was to determine the relationship between allostatic load in ring-tailed lemurs (*Lemur catta*) under human care and potentially stressful individual, social, medical and husbandry factors, as well a sex and age. An allostatic load index (ALI) was calculated for 38 lemurs using six biomarkers measured in serum (albumin, cortisol, dehydroepiandrosterone-sulfate, DNA damage, glucose and prostaglandin E2). Potentially stressful factors were recorded over the lifetime of each lemur using medical and husbandry records. Animals with a higher percentage of time spent indoors, those kept in smaller average group sizes, and those with fewer minor group composition changes had, or tended to have, higher ALI. There was no relationship between ALI and sex or age. Some social and husbandry factors were associated with allostatic load in lemurs, indicating that this index may be a useful tool in assessing and determining factors contributing to stress of lemurs and other animals under human care.

## 1. Introduction

Measuring and mitigating stress in non-human primates is imperative to their welfare. Yet, there are currently few tools for quantifying chronic stress in primates and other animals under our care. In humans, one method used to measure chronic stress uses an estimation of allostasis. Allostasis, defined as the process by which an organism adapts to stressors [1,2] relies on integrated responses of the hypothalamic-pituitary-adrenal (HPA) axis and sympathetic-adrenal-medullary (SAM) axis [3,4] to real and perceived threats to achieve “stability through change”.

Allostatic load (AL) is the cumulative cost to the body of maintaining allostasis [2], or the “wear and tear” imposed on organ systems required to respond to stressors and maintain allostasis. Measurement of allostatic load involves quantifying the biomarkers associated with dysfunction of these organ systems and provides a more comprehensive approach than evaluating a single biomarker to assess stress (e.g., measuring glucocorticoids alone [5]). In humans, an allostatic load index (ALI) has been described to reflect dysregulation in immune, neuroendocrine, cardiovascular and metabolic systems associated with chronic stress [6].

Allostatic load has been evaluated extensively in human populations and has been associated with compromised health and early mortality [7]. While the concept of allostatic load is applied theoretically throughout the animal literature, to date the methodology has only been validated in gorillas (*Gorilla gorilla gorilla*) and rats (*Rattus norvegicus domestica*) [3,8,9,10]. In gorillas, researchers found that ALI was higher in older gorillas, females, and individuals that had experienced more stressful events, such as agonistic interactions with wounding, translocations, and anesthetizations throughout their lifetimes [3]. Moreover, wild-caught gorillas in human care had higher ALI compared with those that were mother-reared [8]. In rats, an ALI referred to as the “rat cumulative allostatic load measure (rCALM)” was found to estimate the effects of chronic stress [10].

No research, to our knowledge, has assessed allostatic load in other non-human primates. Here we describe a study assessing allostatic load in ring-tailed lemurs (*Lemur catta*) housed under human care. Our objective was to determine the relationship between allostatic load index, age, sex, and potentially stressful individual, social, medical and husbandry factors in ring-tailed lemurs.

## 2. Materials and Methods

### 2.1. Animals, Housing, and Inclusion Criteria

The study was conducted using ring-tailed lemurs (*Lemur catta*) housed at the Duke Lemur Center in Durham, North Carolina, USA. All animals were cared for according to Duke University’s Institutional Animal Care and Use Committee (protocol #A027-15-01).

A total of 198 ring-tailed lemurs have been housed at the Duke Lemur Center since its opening. From this group, individuals were selected for inclusion in the study based on the following criteria: (1) the animal is/was >1 year of age at the time of blood collection; (2) medical records with details of health events existed for the individual; (3) Duke Lemur Center is/was the final institution where the animal was held; and (4) there was either 1 mL of banked serum for the individual or they were currently part of the collection and a fresh sample could be obtained. Thirty-eight lemurs met all four of these criteria (16 males and 22 females; age range one–30 years) and were included in the study.

Lemurs kept at the Duke Lemur Center are housed in a variety of settings ranging from indoor enclosures to the semi-free ranging areas where they are primarily outdoors, except during extreme weather events (e.g., cold, snow, hurricanes) when they are shifted into heated indoor enclosures. The larger semi-free ranging enclosures are on 1.5 to 14.3 ha tracts of land. Individuals were fed daily rations of primate diet (Purina**^®^**MonkeyDiet5038, PMI Nutrition International, Inc., Brentwood, MO, USA) supplemented with fresh fruits and vegetables. When lemurs had access to the outdoor semi-free ranging enclosures, they were able to forage from the forest. Clean water was available *ad libitum*.

### 2.2. Serum Sample Collection

Banked serum was available for 27 lemurs included in the study. If banked serum was not available, fresh blood was collected by a veterinarian (*n* = 11). To collect the blood sample, lemurs were captured using a net and moved into an examination room under manual restraint. All blood samples were centrifuged at 2500 rpm for 10 min to extract serum. Serum was kept frozen at −18 °C until analysis. Serum samples were aliquoted into 0.5 mL vials to minimize the need to thaw a sample more than once.

### 2.3. Biomarkers to Estimate Allostatic Load

Biomarkers used to estimate allostatic load index in ring-tailed lemurs were selected based on the following criteria: (1) they had been used in research assessing allostatic load in gorillas [3,8]; or (2) they had been used in human models of allostatic load [4,6]; (3) they were measurable in serum and (4) they were measurable using commercially available assays. Table 1 shows the six biomarkers that met these criteria and were used to calculate allostatic load index in the lemurs.

#### 2.3.1. Immune/Cardiovascular Markers

Albumin is a protein produced by the liver and its transcription is downregulated in the face of trauma, inflammation, neoplasia, or other insult (CUCVM, 2013). Thus, albumin is considered a negative acute phase protein and an immune system marker which decreases during acute inflammation. Albumin has also been shown to be a negative indicator of cardiovascular health in humans and is associated with increased risk of heart failure and cardiovascular disease [11,12]. Prostaglandin E2 (PGE-2) is a principal mediator of inflammation that is synthesized as part of the arachidonic acid cascade [13] and is stimulated by trauma or through signaling molecules. Under normal circumstances PGE-2 has a homeostatic effect in helping the body mediate inflammation.

#### 2.3.2. Neuroendocrine Markers

Cortisol is a glucocorticoid produced by the adrenal gland in response to the stimulation by the HPA axis. Cortisol plays an essential role in the maintenance of most homeostatic functions and is commonly used as a biomarker for stress in free-ranging lemurs [14,15,16]. Dysregulation of the HPA axis can result in prolonged elevation of cortisol or adrenal exhaustion resulting in decreased cortisol production. Dehydroepiandrosterone-sulfate (DHEA-S) is another hormone produced by the adrenal gland that impacts the production of androgens and estrogens. DHEA-S has been shown to have significant impacts on health in humans [17].

#### 2.3.3. Metabolic Markers

Glucose is derived from dietary carbohydrates and is the primary energy source for mammalian cells. The regulation of glucose is mediated by multiple hormones including insulin, catecholamines and glucocorticoids. In the face of stressors, glucose can be elevated either transiently or for prolonged periods, both of which can have adverse health effects [18]. The DNA damage test identifies oxidized guanine species as a measure of cumulative oxidative stress in the body. Oxidative stress is defined as “a disturbance in the balance between the production of reactive oxygen species (free radicals) and antioxidant defenses” [19]. Oxidative stress can cause damage to DNA, leading to modifications that can have pathological consequences such as cancer or Alzheimer’s disease in humans [20,21].

### 2.4. Assays and Assay Validation

Albumin and glucose values were obtained from standard chemistry panels that were included in the medical records on the date of the blood collection. All other biomarkers were measured using competitive binding enzyme linked immunoassays (ELISAs) run in-house after biological validation. All samples were run in duplicate on 96-well plates. Plates were read using a Tecan microplate reader (Tecan Group Ltd., Seestrasse 103, 8708 Männedorf, Switzerland) at a wavelength of 450 nm.

A biological validation approach was taken to ensure that the assays being used to quantify the biomarkers utilized in allostatic load score accurately measured these compounds in ring-tailed lemurs. To determine if each analyte of interest was being measured by the assay, samples were serially diluted to evaluate parallelism with the standard curve.

Intra-assay precision was calculated for cortisol and PGE-2 ELISAs by running three individual’s samples multiple times and determining the % covariance (CV) using the following formula:

%CV = (standard deviation of data-set/Mean of data-set) × 100

Intra-assay precision could not be run for DNA damage or DHEA-s ELISAs due to limited serum volume availability. Inter-assay coefficients of variation were not performed since all assays were run within a 24–48 h period by the same individual.

Competitive ELISAs were run using monoclonal antibodies for the detection of cortisol (NCalTM International Standard Kit, DetectX, Cortisol Enzyme Immunoassay Kit, Species independent, Arbor Assays Interactive Assay Solutions, Ann Arbor, MI, USA), DHEA-S (DetectX**^®^** Dehydroepiandrosterone sulfate (DHEA-S) Immunoassay Catalog # K054-H1, Arbor Assays Interactive Assay Solutions, Ann Arbor, MI, USA), PGE-2 (DetectX **^®^** PROSTAGLANDIN E2 Enzyme Immunoassay Kit catalog #K051-H1, Arbor Assays Interactive Assay Solutions, Ann Arbor, MI, USA). The DNA damage ELISA is a competitive monoclonal assay designed to measure RNA and DNA oxidized guanine species (DetectX**^®^** DNA Damage Immunoassay Catalog # K059-H1, Arbor Assays Interactive Assay Solutions, Ann Arbor, MI, USA). All kits were run following manufacturer instructions.

### 2.5. Allostatic Load Index (ALI) Estimation

An allostatic load index was calculated for each lemur using established methodology in humans [22]. Raw values for each biomarker were divided into quartiles. Albumin and DHEA-S have a greater risk of impairing physiological regulatory mechanisms when they fall in the lowest quartile, whereas DNA Damage, glucose and PGE-2 show increased risk in the highest quartile (Table 1 [3,8]). Cortisol can be either elevated or depressed during chronic stress; thus, a two-tailed cut-off was applied with the highest and lowest 12.5% of values being classified as high risk. Although there are clinical cut-points for some of these biomarkers, these cut-points were not used so that we could include individuals that may appear clinically healthy but have increased allostatic load [23]. Each biomarker within the high-risk quartile for an individual lemur was scored 1; biomarkers not within the high-risk quartile were given a score of zero. Scores were summed for each lemur as a composite allostatic load index (min = 0, max = 6).

### 2.6. Age, Sex and Stress Factors

The sex and the age of lemurs at the time of sample collection were determined from the medical records. Husbandry, medical and social factors that may impact stress were collected from records (referred to as ‘stress factors’). Factors were chosen that were likely to cause disruption of allostasis due to their assumed negative, chronic and/or severe nature. These factors included: (1) anesthetic events, (2) manual restraint, (3) transfers between institutions, (4) enclosure changes, (5) trauma, (6) illness, (7) pregnancy, (8) group composition changes, (9) time spent outdoors in semi-free ranging enclosures, (10) time spent in indoor enclosures, (11) time participating in research projects, and (12) average lifetime group size. Each stress factor was summarized over the lifetime of each lemur before analysis.

Group composition changes were classified as major or minor. Major group structure changes included: (a) first-time separation from generational sibling or dam; (b) the addition of a group of another species into the enclosure: (c) the introduction of two individuals for the first time for breeding purposes; (d) isolation of the focal animal from conspecifics; and (e) a birth in the group. All other group composition changes, including removal or addition of unrelated females or males, were considered minor. In addition to being evaluated separately, a composite total number of group composition changes was also calculated by summing the number of major and minor events.

Research projects were further categorized as either minor or major. Major research events were defined as those requiring direct manipulation of the lemur (e.g., handling or moving to another location); minor events involved changes to the environment with no direct handling. Some research projects included anesthesia; these cases were coded as an anesthetic event and not a research project. Some research projects included blood samplings that were opportunistically collected for medical reasons; these cases were coded as a manual restraint event and not a research project.

Before analysis, we created a variable called ‘total stress factors’ by summing the number of anesthetic events, manual restraints, institutional transfers, enclosure changes, traumas and illnesses; these variables were chosen based on previous work in gorillas [3].

### 2.7. Statistical Analysis

All statistical analyses were completed using SPSS Statistics Base (IBM Corporation, Armonk, NY, USA). Data points were considered extreme outliers if they were three times the interquartile range (IQR) greater than the 3rd quartile (Q3) (e.g., Q3 + 3 × IQR) or three times the IQR less than the first quartile (Q1) (e.g., Q1 − 3 × IQR). No outliers were found. Descriptive statistics were calculated for age and ALI. To ensure that males and females had the same general distribution for each biomarker, two-sample *t*-tests were conducted. No significant differences between males and females for any biomarker were observed, thus the same quartile cut points were used for males and females.

The allostatic load index is an ordinal variable but is commonly normally distributed in humans. ALI was also normally distributed in our sample of lemurs; therefore, linear models were used. Differences in ALI between male and female lemurs were compared using a two-sample *t*-test.

Linear regression was used to determine the relationship between ALI and age, total stress factors, and each individual stress factors (number of anesthetic events, manual restraints, number of institutional transfers, enclosure changes, trauma, illness, pregnancies, group composition changes, percentage of time spent in outdoor vs. indoor enclosures, number of research events/time participating in research, and average group size over a lifetime). If an association between ALI and a stress factor showed a tendency (*p* < 0.10), males and females were compared separately to determine if there was a stronger association within one sex. Differences at *p* ≤ 0.05 were considered significant and 0.05 < *p* ≤ 0.10 were considered a trend.

## 3. Results

### 3.1. Assay Validation

There was parallelism of the serially diluted samples to the standard curve for cortisol, DHEAs, DNA damage, PGE-2 ELISAs. The intra-assay precision for the cortisol ELISAs ranged from 11.8–25.8%, and for the PGE-2 ELISAs it was 18.25%.

### 3.2. Descriptive Statistics for Age and Allostatic Load Index (ALI)

Age of the individual in the sample population ranged from one–30 years, with a median of 11 years and a standard deviation of eight years. In this sample of animals, allostatic load indexes ranged from 0 to 4 out of a possible score of 6. The mean ALI was 1.7 with a standard deviation of 1.0.

### 3.3. Effect of Sex, Age and Stress Factors on ALI

**Figure 1:**
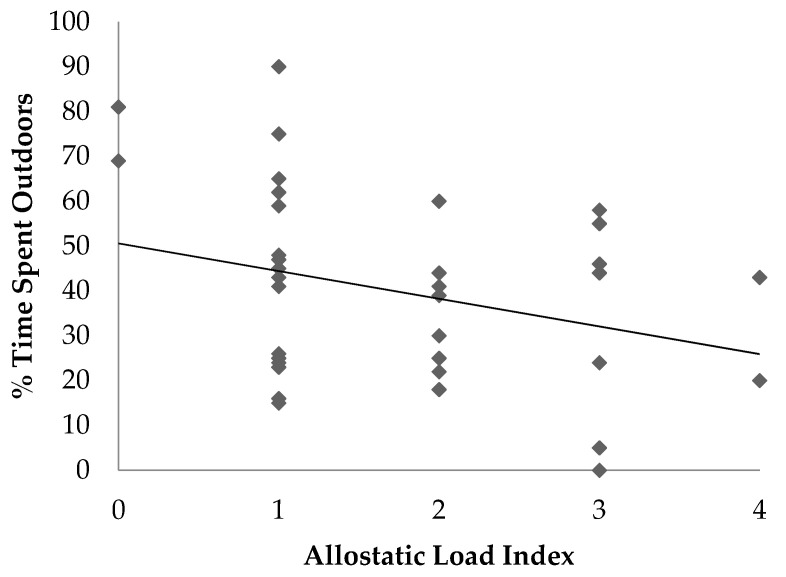
Scatter plot illustrating the association between allostatic load index and the percentage of time an individual spent housed outdoors in ring-tailed lemurs (*Lemur catta*).

There was a tendency for a negative association between ALI and percentage of time spent outdoors in semi-free ranging enclosures (*p* = 0.07, R^2^ = 0.066) (Figure 1). Similarly, there was a positive association between ALI and the percentage of time spent indoors (*p* = 0.05, R^2^ = 0.08).

**Figure 2:**
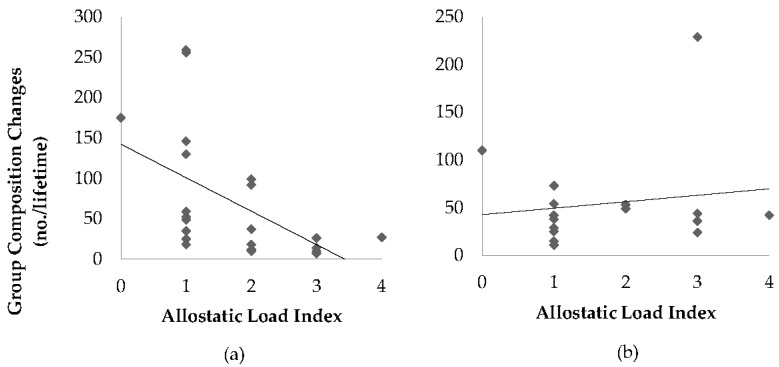
Scatter plot illustrating the association between allostatic load index and the total number of group composition changes experienced by (**a**) female (*n* = 22) and (**b**) male (*n* = 16) ring-tailed lemurs (*Lemur catta*).

There was a tendency for the total number of group composition changes to be related to ALI (*p* = 0.09, R^2^ = 0.05), whereby higher ALI was associated with fewer group composition changes. This tendency was driven by the number of group changes classified as minor (*p* = 0.07, R^2^ = 0.06), but not major (*p* = 0.23, R^2^ = 0.01) group changes. When minor and major group changes were summed and data were analyzed separately by sex, there was a negative association between ALI and total number of group composition changes in females (*p* = 0.01, R^2^ = 0.25), but not males (*p* = 0.59, R^2^ = 0.05) (Figure 2).

**Figure 3:**
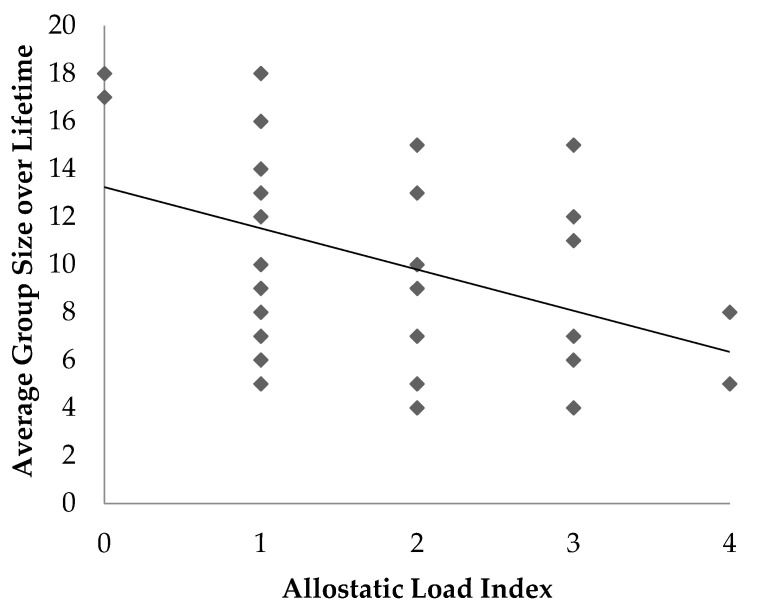
Scatter plot illustrating the association between allostatic load index and the percentage of time an individual spent housed outdoors in ring-tailed lemurs (*Lemur catta*).

There was a negative association between average group size and ALI (*p* = 0.02, R^2^ = 0.13) whereby lemurs with average smaller group sizes throughout their lifetimes had higher the ALI (Figure 3).

There was no effect of age or sex on ALI (sex: *p* = 0.94, t = 0.07, DF = 36; age: *p* = 0.14, R^2^ = 0.04). We found no associations between ALI and total stress factors, number of anesthetic events, manual restraints, institutional transfers, enclosure changes, trauma events, illnesses, pregnancies (females only), or the number of times the animals participated in research studies (Table 2).

## 4. Discussion

The objective of this study was to determine if an allostatic load index was associated with age, sex or factors that may be potential stressors in ring-tailed lemurs housed at the Duke Lemur Center. The results provide several insights into the relationship between ALI and stressors in ring-tailed lemurs. Associations and trends were identified between ALI and some social and husbandry factors, including access to indoor or outdoor enclosures, the number of group composition changes, and average group size.

In this study, the percentage of time each lemur spent indoors was positively associated with allostatic load, and time spent outdoors in semi-free ranging enclosures tended to be negatively associated with allostatic load. There are several potential explanations for these associations. The first is that the outdoor semi-free ranging enclosures are similar to the lemurs’ natural forested habitat which may reduce stress compared with indoor housing. Prior work has shown that naturalistic habitats in captive settings are more suitable than non-naturalistic enclosures and are better at meeting the biological and behavioral needs of the animal [24]. Another potential reason for the lower allostatic load in animals housed outdoors more often may have to do with an increase in control over their environment. In other species, a perceived lack of control and unpredictable husbandry routines are often associated with increased levels of stress hormones and behavioral changes [25,26]. In the semi-free ranging enclosure areas, the lemurs could choose where and with whom to spend their time. This provides the ability to form more natural relationships and exhibit normal behaviors. Lastly, the association between decreased allostatic load and time spent outdoors in semi-free ranging enclosures may be associated with exposure to natural sunlight. In humans and animal species, vitamin D deficiency has been linked to several poor health outcomes [27]. More work would be needed to determine the long-term benefits of semi-free ranging environments for ring-tailed lemurs.

We found a tendency for higher ALI in animals with fewer group composition changes, and this tendency was driven by “minor” group changes and was found in females but not males. One potential explanation for this result is that more frequent changes in the group structure meant that the dominance hierarchy was more often altered, so no one individual was always dominant or always subordinate. Perhaps this allowed for increased fluidity in the group dynamic and decreased the chronic stress on any one individual. Although dominance has been shown to be linked to stress in many primate species, whether the dominant or the subordinate individual experiences the bulk of stress is species-dependent [28,29,30]. In ring-tailed lemurs, dominance is not linear and often changes, making it more complicated to sort out the potential impacts it may have on chronic stress. Lack of an association between ALI and what were classified as major group changes, specifically separation from a dam or generational sibling, was also surprising given the close familial bonds maintained in wild social groups [14,31,32]. This finding may indicate that there are more subtle interactions occurring and that any group composition change could potentially have an impact on allostatic load index and the events that were classified as minor were not.

Average group size was negatively associated with allostatic load; animals that were maintained in smaller groups over their lifetime had higher allostatic load. Lemurs in larger groups may experience more opportunities for positive social relationships with other lemurs compared to those in smaller groups [16]. This finding may also be confounded with outdoor access, as large groups are more likely to reside in the outdoor semi-free ranging area whereas smaller groups are more likely to be housed in smaller indoor enclosures.

We detected no association between ALI and sex or age in the lemurs used in our study. In contrast, research on gorillas reported that females and older gorillas had higher allostatic load compared to males and younger animals [3,8]. In humans, allostatic load has also been found to increase with age and vary by sex [33]. The lack of association between ALI and age in our study may indicate gaps in the allostatic load index or may be due to a lack of variation in age in our sample. Perhaps additional biomarkers need to be validated to construct a more robust estimate of allostatic load in lemurs.

The lack of an association between ALI and sex in our study could be due to methodological and/or species differences. Gorillas have a male-dominant social structure with one silverback and multiple females; while males are always dominant, there is also a hierarchy among females [34]. The presence of a single adult male in each group eliminates the need to compete with other males over females, food, territory, and other resources, therefore possibly leading to a lower ALI in males. In contrast, female gorillas housed in zoos may have higher ALI because they are unable to avoid aggression from either the male or other females in the group [3]. Captive lemur social structure is markedly different than captive gorillas, with animals housed in multi-male, multi-female groups. While a female dominance hierarchy exists, it is disrupted by intense mate competition during the breeding season [32]. Hierarchical fluidity may lead to males and females experiencing chronic stress equally, and thus having similar ALI.

Many of the stress factors, as well as the total stress factors, were not associated with allostatic load index in our study. This result contrasts with research in captive gorillas where a similar total stress score was positively associated with allostatic load [3]. This difference may indicate that ring-tailed lemurs are more flexible or resilient to the stressors measured in this study compared to gorillas, or the stressors we chose to measure were not the most relevant to this species.

Limitations to this study included the retrospective nature of data collection, the sample size, and potential lack of validated biomarkers necessary to measure allostatic load in lemurs. The retrospective nature of the project means that information had to be obtained from medical and husbandry records which are not always complete. The statistical analysis showed several non-significant tendencies, which may reflect the relatively small sample size. Based on our sample size calculations, 38 individuals should have been large enough to reveal associations between ALI and the predictor variable; however, a larger study population may have led to more significant associations. Overall, the allostatic load index used in ring-tailed lemurs, while still in need of refining, appears to show significant associations with chronic stressors and lends insight into the situations that may be perceived as stressful in this species.

Another limitation to this research is the ability to choose appropriate biomarkers to adequately capture if an animal has experienced chronic stress. Choosing appropriate biomarkers reflecting neuroendocrine, metabolic, inflammatory and cardiovascular dysregulation due to stressors on animals is vital to developing an accurate allostatic load index. In human research, biomarkers used to calculate AL are variable and have been developed over decades. Many of the AL indexes in human populations include primary stress biomarkers (e.g., epinephrine, norepinephrine, dopamine) as well as the secondary biomarkers that were explored in our study [5,7,22]. To extrapolate to other species, it is necessary to first evaluate the appropriateness of each biomarker. Biomarkers for this exploratory study were chosen based on previous studies, our ability to assess them from a single serum sample and the availability of commercial assays. Further research into the use of ALI in lemur species is warranted, including the incorporation of primary stress biomarkers.

## 5. Conclusions

Measuring chronic stress in non-human primates is difficult using one biomarker alone. In this study, we took the first step to determine if a combination of biomarkers that reflect allostatic load is related to potential stressors in ring-tailed lemurs. We found that age and sex were not related to allostatic load index in lemurs. Some social and husbandry factors were, or tended to be related to allostatic load index, including the percentage of time spent outdoors, the number of group composition changes, and larger average group sizes. Ongoing work and refinement is needed to determine if these biomarkers can be used to measure chronic stress in ring-tailed lemurs and other animals.

## Figures and Tables

**Table 1:** Biomarkers used in the estimation of allostatic load for in ring-tailed lemurs (*Lemur catta*) and the response of the biomarkers to chronic stress.

Biomarker	Function	Response to Chronic Stress
Albumin	Negative acute phase protein, downregulated in response to inflammation	Decreases
Cortisol	Suppresses inflammation, and induces gluconeogenesis	Increases or decreases
Dehydroepiandrosterone-sulfate (DHEA-S)	Functional hypothalamic-pituitary-adrenal axis (HPA axis) antagonist, suppresses inflammatory cytokines	Decreases
DNA Damage	Measure of oxidative stress and indicative of potential free radical damage.	Increases
Glucose	Main source of metabolic energy	Increases
Prostaglandin E2 (PGE-2)	Part of the inflammatory cascade, associated with neoplasia and Alzheimer’s in humans	Increases

**Table 2:** The relationship between factors that may influence stress in ring-tailed lemurs (*Lemur catta*) and allostatic load index (0 to 6) calculated for each lemur using six biomarkers.

Stress Factor	R^2^	*p*
Anesthetic events (no./lifetime)	0.01	0.25
Manual restraints (no./lifetime)	−0.02	0.64
Institutional transfers (no./lifetime)	0.01	0.27
Enclosure changes (no./lifetime)	−0.01	0.39
Trauma events (no./lifetime)	0.02	0.21
Illnesses (no./lifetime)	−0.01	0.48
Pregnancies (females only; no./lifetime)	0.01	0.54
Major research studies (no./lifetime)	−0.03	0.97
Minor research studies (no./lifetime)	−0.11	0.44
Total stress factors (no./lifetime)	−0.02	0.72

## Data Availability

The data presented in this study are available on request from the corresponding author. The data are not publicly available currently.
